# Reconciling Forest Conservation and Logging in Indonesian Borneo

**DOI:** 10.1371/journal.pone.0069887

**Published:** 2013-08-14

**Authors:** David L. A. Gaveau, Mrigesh Kshatriya, Douglas Sheil, Sean Sloan, Elis Molidena, Arief Wijaya, Serge Wich, Marc Ancrenaz, Matthew Hansen, Mark Broich, Manuel R. Guariguata, Pablo Pacheco, Peter Potapov, Svetlana Turubanova, Erik Meijaard

**Affiliations:** 1 Center for International Forestry Research, Bogor, Indonesia; 2 School of Environment, Science and Engineering, Southern Cross University, Lismore, NSW, Australia; 3 Institute of Tropical Forest Conservation (ITFC), Mbarara University of Science and Technology (MUST), Kabale, Uganda; 4 Centre for Tropical Environmental and Sustainability Science, School of Marine & Tropical Biology, James Cook University, Cairns, QLD, Australia; 5 Research Centre in Evolutionary Anthropology and Palaeoecology, School of Natural Sciences and Psychology, Liverpool John Moores University, Liverpool, United Kingdom; 6 Sabah Wildlife Department, Kota Kinabalu, Sabah, Malaysia; 7 HUTAN, Kinabatangan Orang-utan Conservation Programme, Kota Kinabalu, Sa,bah, Malaysia; 8 North England Zoological Society, Chester Zoo, Chester, United Kingdom; 9 Department of Geographical Sciences, University of Maryland, College Park, Maryland, United States of America; 10 The Climate Change Cluster, University of Technology Sydney, NSW, Australia; 11 Borneo Futures Project, People and Nature Consulting International, Ciputat, Jakarta, Indonesia; 12 School of Biological Sciences, University of Queensland, Brisbane, Australia; Midwestern University & Arizona State University, United States of America

## Abstract

Combining protected areas with natural forest timber concessions may sustain larger forest landscapes than is possible via protected areas alone. However, the role of timber concessions in maintaining natural forest remains poorly characterized.

An estimated 57% (303,525 km^2^) of Kalimantan's land area (532,100 km^2^) was covered by natural forest in 2000. About 14,212 km^2^ (4.7%) had been cleared by 2010. Forests in oil palm concessions had been reduced by 5,600 km^2^ (14.1%), while the figures for timber concessions are 1,336 km^2^ (1.5%), and for protected forests are 1,122 km^2^ (1.2%). These deforestation rates explain little about the relative performance of the different land use categories under equivalent conversion risks due to the confounding effects of location.

An estimated 25% of lands allocated for timber harvesting in 2000 had their status changed to industrial plantation concessions in 2010. Based on a sample of 3,391 forest plots (1×1 km; 100 ha), and matching statistical analyses, 2000–2010 deforestation was on average 17.6 ha lower (95% C.I.: −22.3 ha–−12.9 ha) in timber concession plots than in oil palm concession plots. When location effects were accounted for, deforestation rates in timber concessions and protected areas were not significantly different (Mean difference: 0.35 ha; 95% C.I.: −0.002 ha–0.7 ha).

Natural forest timber concessions in Kalimantan had similar ability as protected areas to maintain forest cover during 2000–2010, provided the former were not reclassified to industrial plantation concessions. Our study indicates the desirability of the Government of Indonesia designating its natural forest timber concessions as protected areas under the IUCN Protected Area Category VI to protect them from reclassification.

## Introduction

Strictly protected areas are established by governments to conserve biological diversity and sustain other values and functions. Extractive and agricultural activities in protected forests are generally prohibited. Most authorities consider that establishing such strictly protected areas represents the best strategy for conserving tropical forests [1]. However, given economic demands, social pressure on land, and the cost of forest protection [2], [3], these areas are unlikely to ever constitute more than a minor part of the tropical landscape, particularly in lowland areas [4], [5], [6]. Some conservation scientists propose combining protected areas with natural forest timber concessions to sustain larger forest landscapes than otherwise possible via protected areas alone [3], [7], [8], [9], [10], [11], [12]. This strategy has the merit of generating income and employment – arguably making it easier to gain political and public support for conservation. The integration of natural forest timber concessions in a forest protection strategy makes sense in countries, such as Indonesia, where protected area management remains weak [13], [14], where the government seeks economic opportunities for its people, and where the urgency of conservation action is high [15].

Natural forest timber concessions are parcels of natural forest leased out to companies or to communities to harvest timber on a long term basis. When natural forest timber concessions are additional to more strictly protected areas they bring an opportunity to maintain larger and better connected forest landscapes with a greater capacity to maintain low density, large range and high mobility species [16]. Indeed, timber concessions are *de facto* a kind of protected area in most tropical countries, as also indicated by their inclusion in the IUCN protected area categories (as Category VI). Conversion of natural forests to plantations in timber concessions is generally prohibited. Concession managers are legally obliged to maintain permanent natural forest cover [9]. Timber harvesting is supposed to be selective [17]. Concession managers only cut the commercially valuable wood above a certain diameter and leave other trees standing for long term regeneration. In equatorial Asia, between two and twenty stems are typically removed from each hectare of forest, once every few decades [18], [19]. Generally, this leaves more than 90% of the trees standing and remaining vegetation recognizably constitutes a forest.

Not only does selective logging maintain a forest structure, a recent global meta-analysis of >100 scientific studies concluded that timber extraction in tropical forests has relatively benign impacts on biodiversity, because 85–100% of mammal, bird, invertebrate, and plant species richness remains in forests that have been harvested once [17]. Thus, a logged tropical forest can remain a biologically rich forest [12]. Not everyone is convinced that natural forest timber concessions should play a major role in tropical forest conservation [20]. Many equate timber harvesting (logging) with forest destruction and loggers with forest destroyers [21], [22]. Many concerns relate to the apparently increased likelihood of a forest harvested for timber being further degraded by wildfires or converted to agriculture. Harvested forests appear to have increased vulnerability to fire [23], [24], [25]. Some governments equate ‘logged forests’ with ‘degraded lands” or “wastelands’, and reclassify these forests for conversion to industrial crops such as oil palm [26]. Roads built to extract timber are also of concern. They increase access which may exacerbate and facilitate illegal encroachments and other threats such as hunting [27], [28], [29], [30], [31], [32], [33]. But, any active timber concession requires people on the ground who might in principle at least enforce regulations and deter illegal activities [12] – thus whether being a timber concessions promotes deforestation compared to other forest land classifications remains debatable.

Despite the interest, the role of timber concessions in maintaining natural forest cover remains poorly characterized. One recent study of all protected areas on the Indonesian island of Sumatra revealed that areas allocated for natural timber harvesting resisted conversion to agriculture as well (or, arguably, as badly) as protected areas during the 1990s [34]. We note that high levels of deforestation sometimes occur in protected areas all over the world [14], [35], [36], [37], but no-one would use this to argue against having protected areas, rather most would suggest that greater efforts should be invested in protection.

Here, we focus on natural forest timber concessions in Kalimantan, the 532,100 km^2^ Indonesian portion of Borneo. Kalimantan is a globally important region for forest biodiversity [38], [39]. Currently, 110,232 km^2^ of Kalimantan's forests are under official protection as national parks, nature reserves and other protected areas. Natural forest timber concessions still make up a large share of Kalimantan's forest landscapes (105,945 km^2^), and include one-third of the habitat of the endangered Bornean orang-utan (*Pongo pygmaeus*) [40]. But, their long-term existence is in jeopardy. As stated earlier, conversion to plantations is prohibited in Indonesian natural forest timber concessions. However, to compensate for the loss of logging revenues following years of harvesting that depleted commercial timber stocks by the late 1980s, the Indonesian government began reclassifying timber concessions in the 1990s into industrial plantation concessions, like monoculture oil palm (*Elaeis guineensis*) and other tree crops such as *Acacia mangium* [41], [42]. Oil palm concessions are parcels of land leased out to companies to establish industrial oil palm plantations. These concessions currently cover 115,500 km^2^ of Kalimantan's land area [43]. If undeveloped oil palm concessions contain natural forests, concession managers are legally obliged to remove these forests to make way for plantations. Usually, the forest is logged first. After all timber resources have been harvested, the remaining trees, shrubs, and debris are often burned. Then, the land is cleared and flattened using heavy machinery to make rows of oil palms. Therefore, reclassification of natural forest timber concessions into oil palm concessions has the immediate effect of legalizing industry-driven deforestation within former timber concessions. During 2000–2010, industrial oil palm plantations in Kalimantan increased from an estimated 8,360 km^2^ to 31,640 km^2^ [43]. Therefore, considering timber concessions as potential protected areas and maintaining their natural forest status could contain the expansion of oil palm into forested areas, and maintain larger and better connected forest landscape with a greater capacity to conserve endangered forest wildlife.

To inform decision-making about the long-term status of natural forest timber concessions, we assessed transitions between official land use categories in Kalimantan (protected area, natural forest timber concession, and oil palm concessions), and studied the change in natural forest cover in each. We compared the total area (248,305 km^2^) set aside for timber harvesting in natural forest (the production zone, or *Hutan Produksi*) by Indonesia's Ministry of Forestry (MoF) in the year 2000 with the area of land allocated for industrial plantations (oil palm and tree crops) and for protection in the year 2010. This production zone includes the 105,945 km^2^ active timber concession licenses mentioned earlier, and areas without active timber licenses. This allowed us to estimate timber concession areas reclassified to protected area and for use as plantations (either oil palm or monoculture tree crops). To test whether natural forest timber concessions (that have not been reclassified to another land use) maintain forest cover, we compared 2000–2010 deforestation rates inside timber concessions with rates inside oil palm concessions; and with rates inside protected areas. Because protected areas tend to be in remote locations and deforestation generally increases with accessibility (e.g. topography) and may also be affected by a variety of other factors, a simple comparison of deforestation rates between logging concession and protected areas would misjudge the protection impact of protected areas [44]. We used “propensity score matching” to help control for and thus reduce any such location dependent biases [14], [45], [46], [47].

## Methods

### Definitions of ‘forest’ and ‘deforestation’ and datasets used

To map deforestation, we used a 60 m^2^ spatial resolution ‘tree cover loss’ map from 2000–2010 generated by authors MH, MB, PP and ST using the methods of Broich et al. [48] and Potapov et al. [49]. ‘Tree cover’ is defined as 60 m^2^ tree stands with >25% canopy cover of ≥5 m in height [48]. ‘Tree cover loss’ is defined as the removal of tree stands. ‘Tree cover’ encompasses any trees including industrial plantations (e.g. oil palm and acacia), mixed traditional gardens (e.g. rubber, orchards, smallholder oil palm and other agro-forests mixed with forest re-growth), as well as old-growth natural forest. ‘Natural forest’ refers to lowland, hill and lower montane dipterocarp forests (often mixed with ironwood stands), mountain forests, freshwater and peat swamp forests, heath forest or *kerangas*, and mangrove forests (including Nipah) [50]. Because we are only interested in the loss of natural forests, we excluded from our analysis all ‘tree cover loss’ pixels (60×60 m) that fell outside of remaining forest areas in year 2000 using a forest cover map generated by Indonesia's Ministry of Forestry (MoF) for year 2000 [51]. The MoF map was created using Landsat images. We assessed its quality by comparing it to our databases of Landsat images. We found that it was in agreement with our independent visual assessment of what constitutes intact natural forests (Primary forest in MoF classification) as well as natural forests degraded by logging, but where the forest remains recognizably a forest (Secondary forest in MoF classification).

### Land use maps

Maps showing the total area set aside for timber harvesting in natural forests (production zone; Hutan Produksi) by the Indonesian government in year 2000 were obtained in 1∶250,000 scale from Indonesia's Ministry of Forestry. Maps of natural forest timber concessions (year 2009–2010) and protected areas (national parks, nature reserves, wildlife sanctuaries, recreational and hunting parks, and watershed protection reserves) were obtained in 1∶250,000 scale from [40], and originate from Indonesia's Ministry of Forestry. Maps of industrial oil palm concession boundaries (year 2005–2008) were obtained from [43], and originate from the provincial governments of Kalimantan. Protected areas created after 2000, for example the Sebangau National Park, were excluded from the propensity score matching analysis.

### Propensity score matching

We tested whether natural forest timber concessions (that were not reclassified to another land use) maintained forest cover during 2000–2010 using propensity score matching. We first generated a sample of homogeneous forest stands, in the form of 100 ha forest plots (1×1 km), which we placed randomly across Kalimantan's 2000 forest cover. Forest plots that were placed within two kilometres of a previously chosen forest plot were rejected. Two kilometres were chosen as a compromise between the need for an adequate sample and the wish to reduce non-independence among observations. From these spatial restrictions, the maximum allowed number of forest plots was n = 6,234 plots. From this sample, only plots that were fully or nearly fully forested (>95 ha in a 100 ha plot) in year 2000 were used to compare deforestation rates between timber concessions, protected areas, and oil palm concessions, to allow the comparison of deforestation amongst plots in number of hectares lost rather than in percentage terms. The final subset retained for this analysis had n = 3,391 plots.

We measured the area of deforestation in each 100 ha plot, with values that ranged from 0–100 ha on a continuous scale, which we considered to be our indicator of effectiveness, and compared the deforestation between plots in timber concession (n = 1,220), in protected areas (n = 1,699), and in oil palm concessions (n = 472).

We used the matching package, *MatchIt* in *R* [52] to control for accessibility dependent effects in deforestation rates and in land use allocation between plots in natural forest timber concessions, protected areas, and oil palm concessions. Based on the literature of tropical deforestation, the variables that best characterize accessibility are slope, elevation above sea level, distance (expressed as travel time) to roads, and to cities [53]. Methods used to extract travel times can be found in File S1. In the context of expanding oil palm plantations in Kalimantan we added distance to oil palm mills, and to existing oil palm plantations in year 2000. These six variables were defined as “control variables” (Figure S1 in File S1). A propensity score was defined as the probability of a 100 ha plot being assigned as a timber concession. This probability was obtained from a logistic regression model in which the presence or the absence of a timber concession in the landscape was regressed against the control variables. The nearest neighbor with caliper procedure was implemented in the *MatchIt* package [52].

For every plot inside timber concessions, *MatchIt* paired up (matched) a plot inside protected areas (or inside oil palm concessions) that possessed the nearest propensity score. No plot could be matched to more than one other plot (without replacement). Only pairs where the difference in propensity scores did not exceed the caliper width were retained. A narrow caliper width was set to 0.25 times the standard deviation of the propensity scores. This narrow caliper width succeeded in matching more similar sites (e.g. protected area and concession plots of similar elevations and slopes) but with fewer number of pairs, thereby increasing the variance of the estimated treatment effect [54]; i.e. the mean difference in deforestation rate. *MatchIt* further restricted the matching across the landscape, so that a matched plot inside a protected area (or inside an oil palm concession) fell within the same administration and within the same soil type as the timber concession plot. This step was taken to ensure that pairs possessed similar socio-ecological and soil characteristics by being not too distant from each other. For example, wildfires are an important driver of deforestation in Eastern Kalimantan, but not in Western Kalimantan [25], [55]. Therefore, matching within the same administration ensures that a plot inside a protected area (or inside an oil palm concession) from Eastern Kalimantan is not matched with a timber concession plot from Western Kalimantan. Eight different administrative groups (*n* = 8) were considered (Figure S1 in File S1). Peat soils and mineral soils were considered because deforestation patterns differ on peat lands; for example industry-driven deforestation for oil palm tends to avoid peat lands in favour of mineral soils [43]. The performance of our matching procedure was evaluated by investigating whether differences in the control variable between pairs had been eliminated [56]. Kolmogorov−Smirnov test (KS-test) and balance statistics provide a way to assess the quality of the matching method [52]. Both methods provide a measure of the balance between the treated and control group before and after matching. The *balance statistic* is a measure of the percent improvement in balance and is defined as 100*((|*a*|-|*b*|)/|*b*|), where *a* and *b* are measures, such as median, mean or maximum, of the original and matched data set respectively [52]. Here, the measures used to compare the un-matched and matched data sets included the empirical quantile median (eQQMedian), mean (eQQ Mean), and maximum (eQQ Max).

## Results

**Figure 1:**
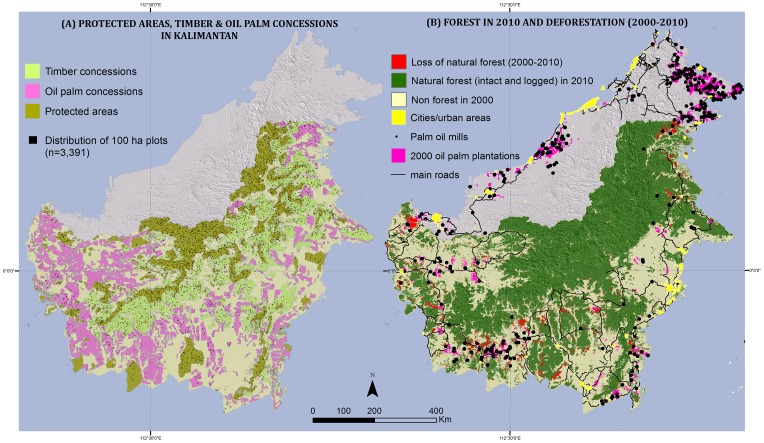
Panel A: protected areas (110,232 km^2^; brown), timber concessions (105,945 km^2^; light green), and industrial oil palm plantation concessions (115,500 km^2^; pink) in 2010 for Kalimantan (532,100 km^2^), and the spatial distribution of the 3,391 forest plots (100 ha each; black boxes). Panel B: remaining forest in 2010 (dark green), deforestation from 2000–2010 (red), main roads (black lines), realized oil palm plantations in 2000 (purple), urban areas (yellow) and palm oil mills (black dots).

The forest cover map generated by Indonesia's Ministry of Forestry indicates that 57% (303,525 km^2^) of Kalimantan's area (532,100 km^2^) was covered in natural old-growth forests (either intact or logged) in 2000. By 2010, this forested area had decreased by 14,212 km^2^, representing a 4.7% loss over the decade. In 2000, the combined area of protected areas and timber concessions contained about 55% (182,185 km^2^) of Kalimantan's natural forests (Figure 1A&B). In the subsequent 10-year period, natural forests occurring in protected areas had been reduced by 1,122 km^2^, representing a 1.2% loss. Forests in timber concessions had been reduced by 1,336 km^2^, representing a 1.5% loss (Table 1). Forests in areas granted to oil palm concessions had been reduced by 5,600 km^2^, representing a 14.1% loss.

**Table 1:** Kalimantan-wide losses in forest cover from 2000–2010.

	Kalimantan	Protected Areas	Timber concessions	Oil palm concessions	Other areas*
**Landmass (km^2^)**	532,100	110,232	105,945	115,500	200,423
**2000 forest cover (km^2^)**	303,524	93,834	88,351	39,722	81,617
**Deforestation (km^2^)**	14,212	1,122	1,336	5,600	6,155
**Deforestation (%)**	4.7	1.2	1.5	14.1	7.5

* Other areas include areas outside of Timber and oil palm concessions and outside of protected areas.

**Figure 2:**
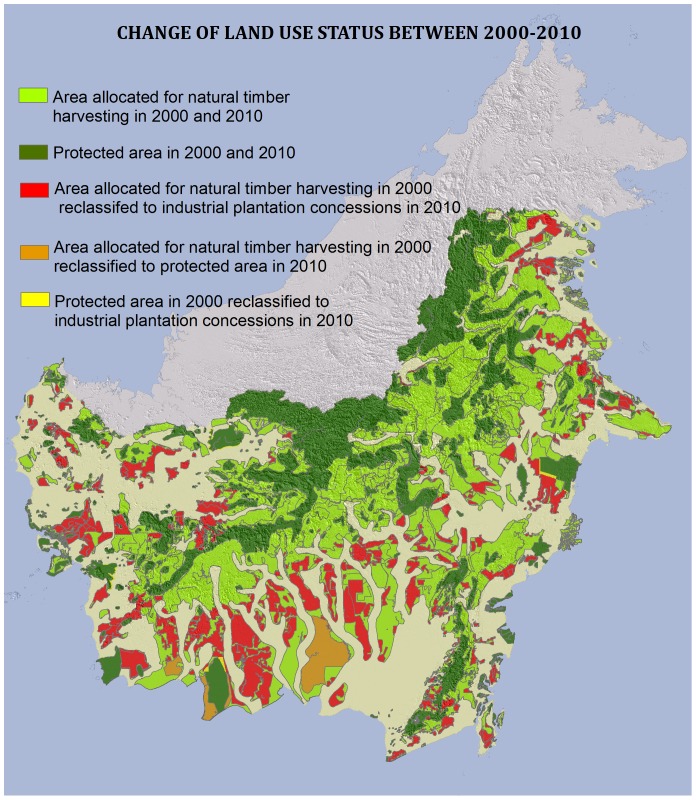
Map showing the change of land use status of area allocated for natural timber harvesting and protected areas during 2000–2010 in Kalimantan. Area allocated for natural timber harvesting in 2000 and 2010 (light green); Protected area in 2000 and 2010 (dark green); Area allocated for natural timber harvesting in 2000 reclassified to industrial plantation concessions in 2010 (red); Area allocated for natural timber harvesting in 2000 reclassified to protected area in 2010 (orange); Protected area in 2000 reclassified to industrial plantation concessions in 2010 (yellow).

The total area (248,305 km^2^) set aside for timber harvesting in natural forests (production zone; *Hutan Produksi*) by the Indonesian government in 2000 had shrunk by 25% by 2010 (Figure 2). The production zone includes the active timber concession licenses (the 105,945 km^2^ area mentioned in Table 1 and shown in Figure 1A), and areas without active timber licenses. An estimated 63,000 km^2^ of the production zone were reclassified to industrial plantation concessions (oil palm and tree crop concessions), while 7,351 km^2^ (3%) were reclassified to protected areas (primarily through the creation of Sebangau National Park). In contrast, less than 1% of protected areas in year 2000 had become reclassified to either natural timber or plantation concessions (Figure 2).

The spatial distribution of our 100 ha plots (n = 3,391) reveals the relative locations of protected areas, timber, and oil palm concessions (Table 2&3). Protected forest plots are typically located in the most remote areas (mean elevation = 636 m; mean slope = 24%; mean travel time to roads, cities, mills and existing plantations >58 hrs; Table 2). Forest plots in oil palm concessions are generally located in the least remote areas (mean elevation = 91 m; mean slope = 4.6%; mean travel time to cities, to mills and existing plantations<18 hrs; Table 3). Forest plots in timber concessions are located in intermediate locations, neither as remote as protected areas or as accessible as oil palm concessions (mean elevation = 360 m; mean slope = 17%; mean travel time to cities, to mills and existing plantations<44 hrs; Table 2&3).

**Table 2:** Summary of balance of the control variables before and after matching for Protected Area (PA) and natural forest Timber Concession (TC) plots.

Variable		Means in TC cells	Means in PA cells	SD Control	Mean Diff	eQQ Med	eQQ Mean	eQQ Max
**Travel time to cities (hr)**	**Before**	43.1	61.9	45.8	−18.8	18.1	18.7	37.6
	**After**	48.9	56.6	41.7	−7.8	7.9	7.8	26.3
	**% Balance Improvement^a^**				61.9%	61.1%	61.8%	40.6%
**Travel time to mills (hr)**	**Before**	42.4	64.6	44.5	−22.2	23.9	22.2	40.0
	**After**	49.4	55.5	41.4	−6.1	4.2	6.1	37.0
	**% Balance Improvement^a^**				74.1%	88.0%	74.0%	17.6%
**Travel time to roads (hr)**	**Before**	41.7	59.0	42.3	−17.3	18.7	17.2	33.6
	**After**	48.0	55.1	41.0	−7.1	7.2	7.1	16.8
	**% Balance Improvement^a^**				62.3%	68.2%	62.2%	53.6%
**Travel time to plantations (hr)**	**Before**	39.6	63.6	45.0	−24.0	26.0	24.0	44.3
	**After**	47.4	54.0	40.5	−6.6	6.3	6.6	22.4
	**% Balance Improvement^a^**				74.8%	81.6%	74.7%	51.3%
**Elevation (m)**	**Before**	359.7	636.4	400.5	−276.6	326.7	282.8	439.4
	**After**	453.5	505.0	312.5	−51.5	65.6	66.6	371.9
	**% Balance Improvement^a^**				82.6%	80.8%	77.6%	14.1%
**Slope (percent)**	**Before**	17.1	24.2	13.2	−7.1	7.7	7.3	30.9
	**After**	20.5	21.8	12.0	−1.3	1.1	1.4	11.1
	**% Balance Improvement^a^**				80.1%	81.3%	79.9%	87.0%

**Table 3:** Summary of balance of the control variables before and after matching for natural forest Timber Concession (TC) and Oil Palm Concession (OPC) plots.

Variable		Means in OPC cells	Means in TC cells	SD Control	Mean Diff	eQQ Med	eQQ Mean	eQQ Max
**Travel time to cities (hr)**	**Before**	17.6	43.1	37.1	−25.5	19.4	26.2	98.1
	**After**	24.8	28.5	27.9	−3.8	11.6	12.9	99.9
	**% Balance Improvement^a^**				90.5%	48.5%	51.1%	−4.4%
**Travel time to mills (hr)**	**Before**	17.6	42.4	34.7	−24.8	18.1	25.9	93.7
	**After**	24.9	27.1	25.5	−2.3	9.3	12.2	107.1
	**% Balance Improvement^a^**				95.3%	51.1%	53.7%	−12.4%
**Travel time to roads (hr)**	**Before**	16.4	41.7	37.0	−25.3	18.5	26.0	101.2
	**After**	22.1	26.9	28.1	−4.8	11.4	12.6	96.0
	**% Balance Improvement^a^**				86.0%	46.3%	51.6%	2.4%
**Travel time to plantations (hr)**	**Before**	13.9	39.6	35.3	−25.7	16.4	26.3	92.3
	**After**	20.4	24.4	26.1	−4.1	8.2	12.2	94.5
	**% Balance Improvement^a^**				87.4%	53.0%	53.9%	4.0%
**Elevation (m)**	**Before**	90.8	359.7	292.4	−268.9	201.6	269.0	888.8
	**After**	164.1	167.5	162.3	−3.5	20.0	29.1	235.0
	**% Balance Improvement^a^**				98.0%	91.0%	89.6%	65.1%
**Slope (percent)**	**Before**	4.6	17.1	11.7	−12.4	12.9	12.5	22.6
	**After**	8.5	8.4	8.5	0.2	0.5	0.8	11.3
	**% Balance Improvement^a^**				98.9%	97.1%	91.6%	31.9%

To control for such location specific effects in our comparison of deforestation rates *Matchit* selected 575 pairs for the logging concession versus protected area analysis and 194 pairs for the logging concession versus oil palm concessions analysis.

**Figure 3:**
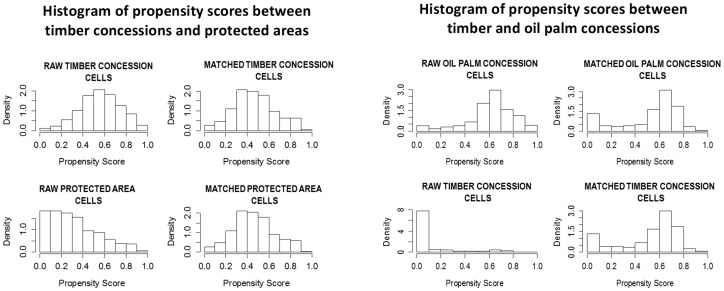
Histogram distribution of propensity scores before and after matching between timber concessions and protected areas (left panel); and between timber and oil palm concessions (right panel).

The distribution of propensity scores between timber concessions and protected areas differed significantly before matching (KS-test for the “raw” dataset: D = 0.4966, p-value<0.001) and did not differ significantly after matching (KS-test for “matched” dataset: D = 0.0313, p-value = 0.9408, Figure 3A). The distribution of propensity scores between timber concessions and oil palm concessions differed significantly before matching (KS-test for the “raw” dataset: D = 0.6431, p-value<2.2e-16). After matching these differences disappeared (KS-test: D = 0.0309, p-value = 1.00) (Figure 3B).

For all control variables, the mean difference (“Mean diff”) decreased after matching as indicated by the balance indices (Table 2&3). The various measures used to gauge departure from perfect matching, such as the empirical quantile median (eQQMedian), mean (eQQ Mean) and maximum (eQQ Max), showed a common trend. The mean magnitudes of each of these statistics became smaller indicating that the matching had resulted in very similar distributions of all the variables considered. These indicators of good matching give us more confidence that the differences in deforestation we observe among the different land use categories can be attributed to their official status rather than to other factors.

**Figure 4:**
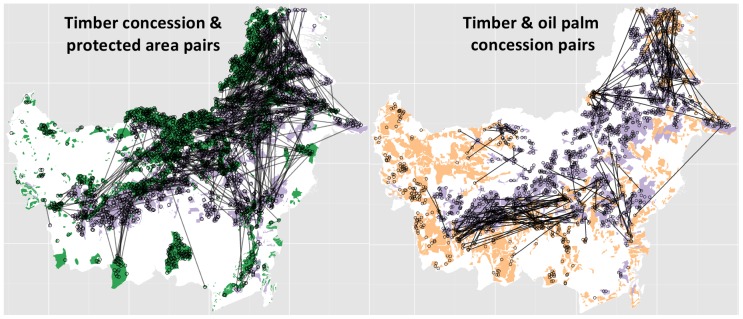
The spatial distribution of the 575 pairs for the natural forest timber concession (purple) *versus* protected area (green) analysis (left panel). The spatial distribution of the 194 pairs for the natural forest timber concession (grey) *versus* oil palm concessions (orange) analysis (right panel).

Based on the unmatched sample dataset mean differences in deforestation from 2000–2010 (expressed in hectares lost in 100 ha plots) are all significant (Table 4). After matching, the mean deforestation was still significantly 17.6 ha lower in timber concessions than in oil palm concessions (95% C.I.: −22.3 ha–−12.9 ha; Table 4). Most importantly, any difference in deforestation rates between natural timber concessions and protected areas was smaller than could formally be detected using this method meaning that there is little difference (mean difference: 0.35 ha; 95% C.I.: −0.002 ha–0.7 ha). The spatial distribution of the pairs is shown in Figure 4.

**Table 4:** Comparison of mean differences in deforestation (2000–2010) before and after matching.

	TC *vs* OPC	TC *vs* PA	TC *vs* managed PA
**Mean Deforestation rates before matching (ha)**	0.91 *vs* 22.21	0.91 *vs* 0.16	0.91 *vs* 0.19
**Mean difference before matching (ha)**	−21.3	0.75	0.72
**(95% C.I.)**	−24.8–−18.5	0.43–1.05	0.33–1.11
**Number of 100 ha plots**	1220 *vs* 472	1220 *vs* 1699	1220 *vs* 594
**Mean difference after matching (ha)**	−17.6	0.35	0.66
**(95% C.I.)**	(−22.3–−12.9)	(−0.002–0.7)	(−0.11–1.43)
**Number of paired 100 ha plots**	194	575	111

These values are expressed in hectares lost in 100 ha plots that were nearly fully forested (>95 ha forest cover) in year 2000. Values ranged from 0 ha lost to 100 ha lost on a continuous scale. Confidence intervals for the unmatched dataset are derived from an independent samples t-test. Confidence intervals for the matched dataset are derived from the matching algorithm, *MatchIt*. The mean difference is between: (i) Timber Concession plots (TC) and Oil Palm Concession plots (OPC); (ii) Timber Concession plots (TC) and Protected Area plots (PA) ; and Timber Concession plots (TC) and managed Protected Area plots (i.e. national parks and nature reserves, but excluding watershed protection forests which are generally not managed).

The protected area category included >50% watershed protection forest reserves (*Hutan Lindung*, HL), areas that, except for a few exceptions of locally funded watershed areas, receive neither funds nor are actively managed by governmental agencies. By grouping these HL reserves with protected areas designated for their conservation values (e.g. national parks), the above analysis potentially diluted the protection impact of managed protected areas. However, when HL reserves were excluded from the protected area category, deforestation was still not significantly higher in natural forest timber concessions than in protected areas (mean difference: 0.66 ha; 95% C.I.: −0.11 ha–1.43 ha; Table 4). The distribution of propensity scores between timber concessions and managed protected areas is shown in Figure S2 in File S1. The spatial distribution of the pairs for the timber concession and managed protected areas is shown in Figure S3 in File S1.

## Discussion

This study reveals that Kalimantan's natural forest timber concessions, i.e. parcels of natural forest leased out to companies to extract timber on a long term basis (>30 years), have as far as we are able to determine with available data and controlling for the influence of location, maintained forest cover just as well as protected areas during the 2000–2010 decade, and have prevented government-sanctioned deforestation; illegal forest conversion to industrial oil palm plantations was marginal within timber concessions. These results corroborate findings in Sumatra where areas allocated for natural timber harvesting (production forest) have been found to resist illegal forest conversion to agriculture as well as protected areas during the 1990s when matched to reduce location specific effects [34]. Thus it appears that timber concessions could be used as a conservation intervention to protect tropical forests. These observations come with caveats.

Firstly, we highlight that our results reflect a statistical conclusion: that is that we cannot detect any significant difference in the deforestation rates in protected areas and in timber concessions when we account for location. These results do not mean that these rates are equal, only that any differences are relatively small compared with our ability to detect them unambiguously. For example if our null hypothesis was that timber concessions maintained a 50% higher deforestation rate than protected areas under similar spatial contexts we would not have been able to reject that either. So, substantial uncertainties remain. Despite our use of propensity score matching we recognize that these methods are only an approximate solution and that ambiguities remain regarding the variables considered, their measurement, their spatial correlations and the choices made to control for these – this is an area where we would hope to make further methodological investigations in the future in order to improve confidence and better understand how spatial context influences the probability and extent of forest cover loss.

Secondly, as our analysis shows, between 2000 and 2010, the Government of Indonesia reclassified 25% of areas allocated for natural timber harvesting for use as monoculture oil palm and tree crop plantations. In the same period, the government only reclassified 3% of timber concessions to the status of protected area, primarily through the creation of Sebangau National Park in Central Kalimantan. Although timber concessions areas are officially required to keep a permanent forest cover, their classification seems easily changed and reclassification into industrial plantation concessions legalize deforestation. In contrast, less than 1% of protected areas had their status changed to industrial plantation concessions. Thus, compared to protected areas, timber concessions have been more vulnerable to official reclassification that permits forest conversion. We only expect timber concessions to maintain forest cover if they are not reclassified for plantations. This is a crucial point because the Indonesian government tends to equate ‘logged’ with ‘degraded/wasteland,’ but as research shows, logged forests can still be extremely valuable habitats for orangutans and other species [16], [40], [57], [58]. The creation of the 5,686 km^2^ Sebangau National Park in 2004, an area logged throughout the 1990s, but containing the largest contiguous orangutan population on Borneo [41], indicates that Government of Indonesia is beginning to recognize the value of logged forests for biodiversity conservation.

Despite the legal protection of forests in protected areas and natural forest timber concessions, both land use types lack the management required to prevent all wild fires and illegal agricultural encroachments by small farmers. This situation is not unique to Kalimantan. There is ample evidence that deforestation persists within protected areas because drivers of deforestation, are coupled with a limited protection capacity that largely reflects insufficient management resources [35], [59], [60], [61], [62], [63], [64], [65]. Several studies have shown that protected area management in Indonesia is insufficiently effective to abate threats of deforestation, and in particular fire, illegal logging, and illegal encroachment. For example, Kutai National Park in East Kalimantan province was severely damage by prolonged drought and wildfires in 1982–1983 [66]. Gunung Palung National Park in West Kalimantan province was the site of widespread illegal logging during the early 2000s, following an era of breakdown in law and order [13]. Bukit Barisan Selatan National Park, in southern Sumatra suffered massive deforestation through agricultural encroachment by small famers for coffee plantations [60], [67]. One reason is insufficient funding. In 2006, Indonesia's terrestrial protected areas received an average USD 1.56/ha in government funding and an estimated USD 0.67/ha in funding from non-governmental organizations and international donor agencies [68]. This is considerably lower than the average USD 13 spent on protected area management in countries in the Asia-Pacific Region [69], [70]. The shortfall in Indonesia's protected area funding – that is the funds needed to achieve what their mandate requires –was estimated at US$ 81.94 million for 2006 [68]. Funding allocation and management choices may have further reduced effectiveness. Data are lacking, but claims have been made that those protected areas involving long-term collaboration between non-governmental organizations (NGOs) and park authorities have been more successful in maintaining forest cover [71].

Our findings indicate that both natural forest timber concessions and protected areas have slowed forest cover loss in Kalimantan in the face of expanding plantations. Timber concessions typically generate a higher per hectare revenues than neighboring protected areas. Timber harvesting in natural forests provides one way in which forest lands can provide income and employment while retaining forest: in simple terms, the forest can pay for its own protection. In addition, studies of the perception of people in Kalimantan about the value of forests for their health, culture, and livelihoods show that logged forests remain important for them [72], [73], [74], [75].

We note that significant forest conservation efforts in Indonesia have been focused on generating and enforcing strictly protected areas. There is little doubt that the reclassification of timber production forest to plantations has been facilitated by the pervasive judgment that equates logged forests with “degraded” or “secondary” undeserving of conservation concern. If we started to pay greater attention to the value of logged forest the protection gains may have been even better. Policy makers, officials and concession staff can all be encouraged to take pride in the value of well managed logged forests and their global conservation values.

Our study indicates the desirability of the Government of Indonesia designating its natural forest timber concessions as protected areas under the IUCN Protected Area Category VI, because they perform as effectively as protected areas in maintaining forest cover and should be protected from reclassification. The World Database of Protected Areas contains many examples of permanent forest reserves where hardwood extraction is one of the activities. Adding Kalimantan's natural forest timber concessions to the protected area network would increase the permanently protected forest in Kalimantan by 248,305 km^2^, i.e., the area of production forest that legally should remain forested. Such changes would require a shift in mindset from producers, government, and also conservation groups, especially because government policy presently does not guarantee timber concession permanent status as natural forest. Still, making such a political decision and implementing it accordingly would have long-term benefits for wildlife and the maintenance of ecosystem services from forests, while continuing the generation of income from forests. We note that such changes are required to achieve sustainable forestry practices, which has long been the stated goal of the Ministry of Forestry and such a permanent and inviolate forest estate would certainly also have value under the future of Reducing Emissions from Deforestation and Degradation (REDD) programs in which Indonesia receives payments for reduced forest loss and damage.

Indonesia's government is taking steps towards the long-term maintenance of its natural forests. In recognition of the importance of natural forest timber concessions for biodiversity, economic development, and social aspirations, the government launched the Ecosystem Restoration concept in 2007 [76]. The ecosystem restoration license is granted to companies for a period of 60 years and can be extended once for a further 35 years. The aim of such licenses is to allow heavily harvested forests to recover their potential to produce commercial timber while maintaining a minimum level of ecosystem services, such as biodiversity conservation. The initiative has had a slow start, however, and as of 2012, only 1,005 km^2^ in two areas, or about 0.9% of Kalimantan's total concession area, had been granted an ecosystem restoration license [77].

A major impediment to the permanent protection of natural forests in Kalimantan is the high economic potential of oil palm plantations [43]. The returns on plantations are much higher than returns from timber harvesting in natural forests. The conversion of logged forests to plantations makes economic sense. What may be overlooked in the political decision-making regarding such land use conversions are the significant values of natural forests to the well-being of many of Kalimantan's people [72], [74], [75], [78]. This does not only include people living close to these forests, but also the many people in downstream and coastal areas that are affected by the negative environmental impacts (air pollution, temperature increases, changed flooding regimes etc.) from unsustainable land use [72]. For all the benefits that plantations bring to people, poor accounting of negative impacts impairs political decision-making that maximizes the well-being of Kalimantan's people. Therefore, considering the importance of natural forest timber concessions for biodiversity conservation as well as societal aspirations, and the high rate at which these forests are reclassified to plantations, it seems important that the Government of Indonesia minimize conversion of natural forests to plantations and expand forest restoration opportunities.

## Conclusion

Current policies in Indonesia allow logged forests in natural forest timber concessions to be managed for rehabilitation and ecosystem restoration, or to become converted to industrial plantations. The systematic reclassification of timber concessions to plantations should be prevented. Encouraging rehabilitation and restoration, and discouraging conversion of logged forest could play a big role in helping protect forests and wildlife in Indonesia. If Kalimantan's forests are approximately as well protected from illegal encroachments as they are in protected areas, as our analysis shows, the Indonesian government would do well strategically to commit to keep natural forest timber concessions in production over the long term alongside the protected area network to collectively conserve over two-third of Kalimantan's remaining forests, while at the same time providing income and employment. This could be achieved by reclassifying natural forest timber concessions as protected areas under the IUCN Protected Area Category VI. Such a permanent forest estate offers benefits for biodiversity conservation and other environmental benefits as well as for providing a foundation for further investment in sustainable forestry.

## Supporting Information

File S1 **Supporting information describing how control variables were derived.** This file includes Figure S1, Figure S2, and Figure S3.
(DOC)
