# Supplementary material for: Reconciling Forest Conservation and Logging in Indonesian Borneo
Source: PLoS One. 2013 Aug 14;8(8):e69887. doi: 10.1371/journal.pone.0069887 (PMC3743885; doi:10.1371/journal.pone.0069887)
Supplement: File S1 — Supporting information describing how control variables were derived. This file includes Figure S1, Figure S2, and Figure S3. (DOC) [file pone.0069887.s001.doc]

**File S1. Supporting Information**

**Control Variables**

A digital elevation model from the National Aeronautics Space Administration’s Shuttle Radar Topography Mission (NASA SRTM) was used to generate elevation and slope maps [1]. Data for existing road networks were obtained from the Ministry of Public Works (*Perkerjaan Umum*). Oil palm plantations in yr 2000 were manually digitized using landsat imagery. The location of oil palm mills for Kalimantan were made available at ([http://www.regulations.gov/#!searchResults;rpp=10;po=0;s=epa-hq-oar-2011-0542%257CWilmar](http://www.regulations.gov/#!searchResults;rpp=10;po=0;s=epa-hq-oar-2011-0542%257CWilmar)) and digitized in Arcmap by Janice Ser Huay Lee.

Accessibility of the forest was modelled as travel times rather than straight-line distances [2], in order to simulate people on foot walking along the path of least resistance. Travel times are a function of slope, and a GIS algorithm (Spatial Analyst, ARCGIS 10) was used to generate a friction map from the slope map. Slope dependent off-road walking speeds were based on those calculated for a complex agricultural landscape at the forest margin in the Philippines [2], and it was assumed that off-road speeds in forests were similar to those in complex agricultural landscapes. These data were combined to generate travel time maps to roads, cities, OP mills and border of plantations.


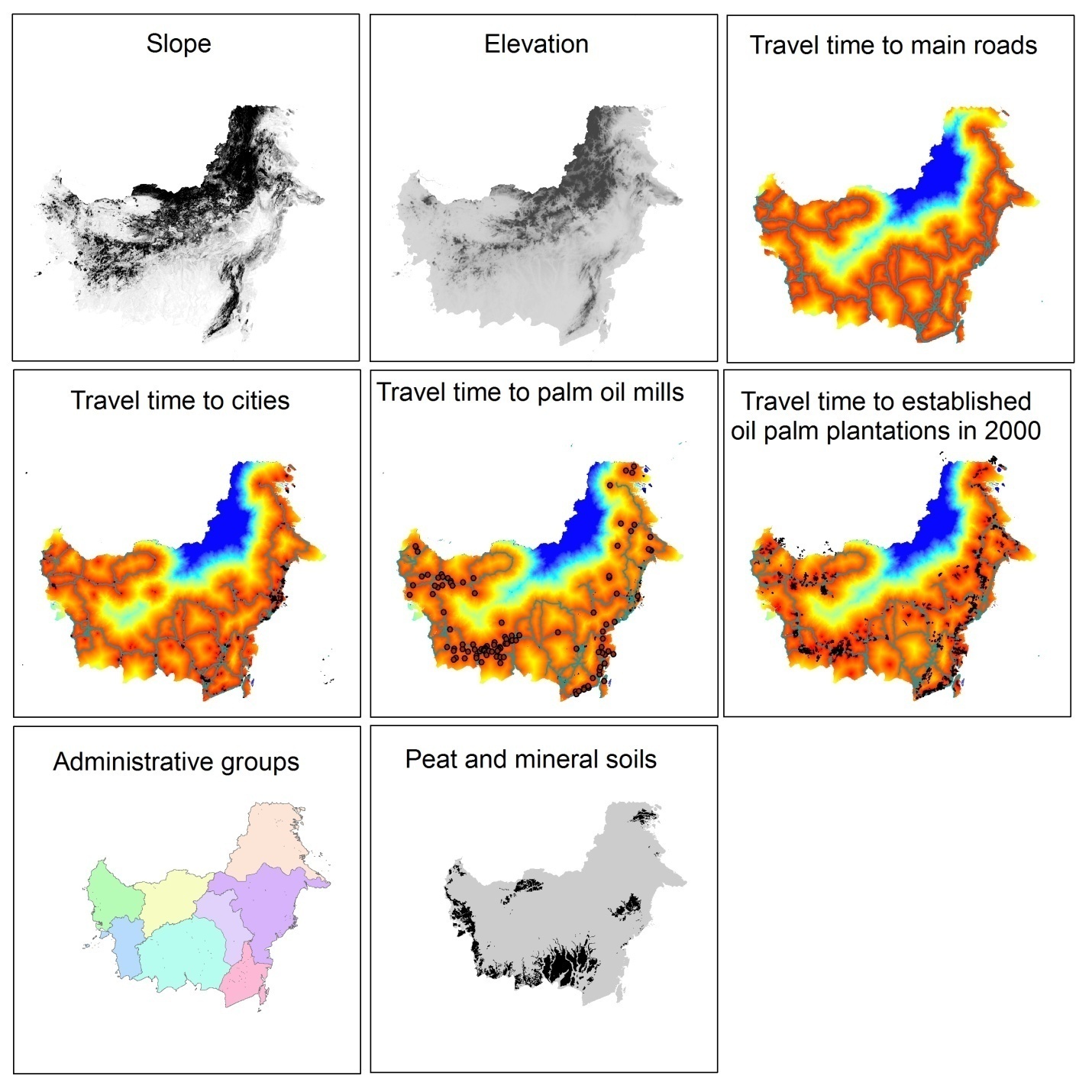


**Figure S1.** The spatial distribution of the control variables


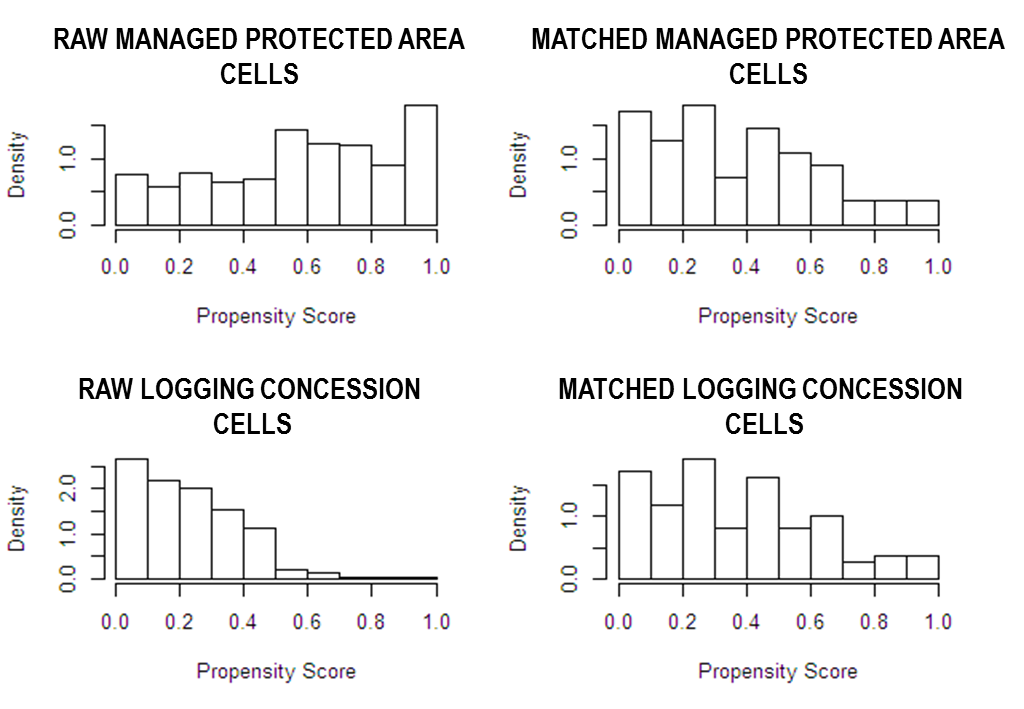


**Figure S2.** Distribution of propensity scores before and after matching between timber concessions and managed protected areas.


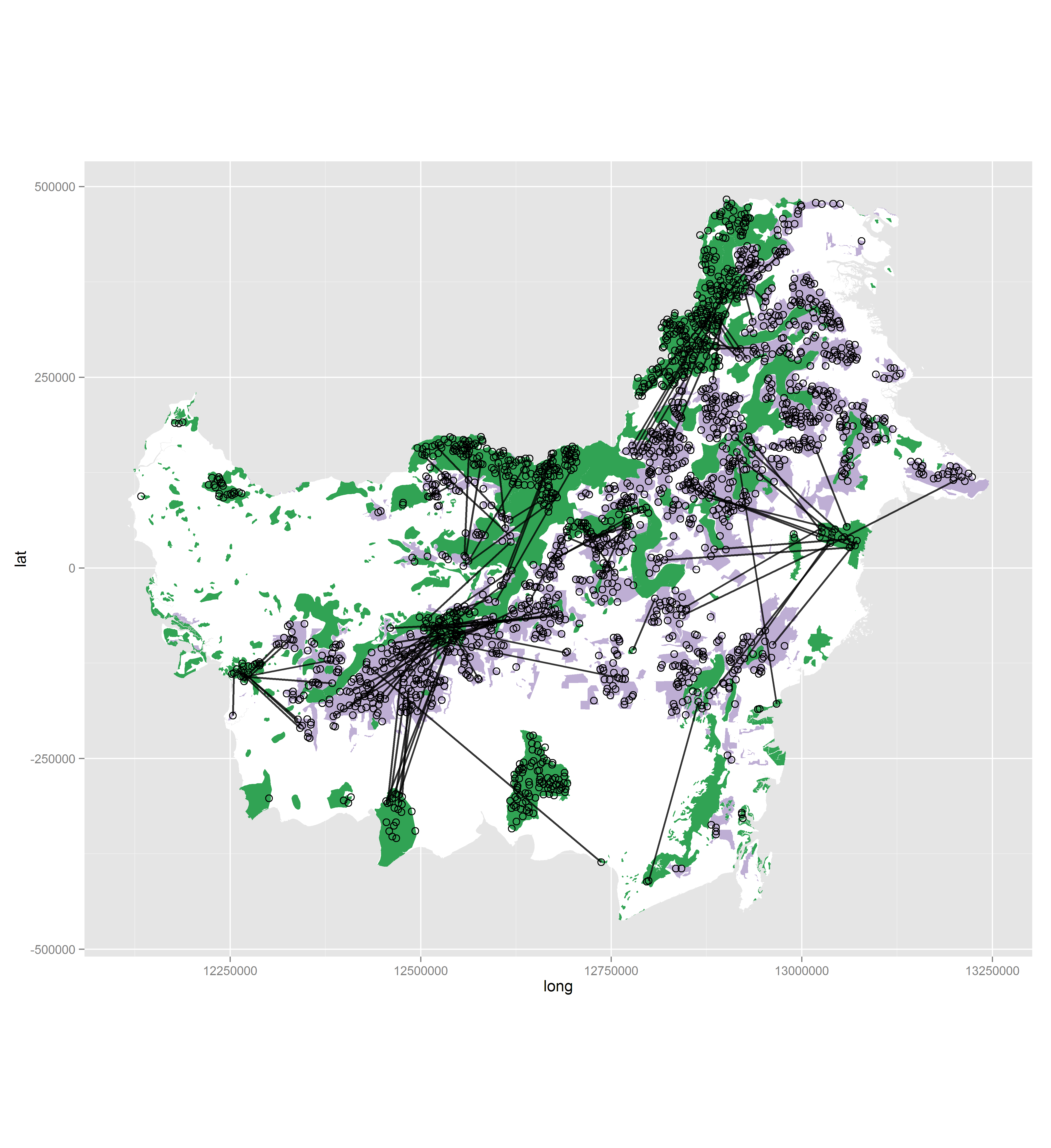


**Figure S3.** The spatial distribution of the 111 pairs for the timber concession (purple) *vs* managed protected area (green) analysis.

1. Rabus B, Eineder M, Roth A, Balmer R (2003) The shuttle radar topography mission-a new class of digital elevation models acquired by spaceborne radar. Photogrammetry and Remote Sensing 57: 241-262.

2. Verburg PH, Overmars KP, Witte N (2004) Accessibility and land-use patterns at the forest fringe in the northeastern part of the Philippines. Geographical Journal 170: 238-255.
